# Heteromorphic stamens are differentially attractive in *Swartzia* (Fabaceae)

**DOI:** 10.1093/aobpla/plac041

**Published:** 2022-09-09

**Authors:** João Paulo Basso-Alves, Rafael Ferreira da Silva, Gabriel Coimbra, Suzana Guimarães Leitão, Claudia Moraes de Rezende, Humberto Ribeiro Bizzo, Leandro Freitas, Juliana Villela Paulino, Vidal de Freitas Mansano

**Affiliations:** Programa de Pós-Graduação em Botânica, Escola Nacional de Botânica Tropical, Jardim Botânico do Rio de Janeiro, Rio de Janeiro, RJ 22460-036, Brazil; Instituto de Pesquisas Jardim Botânico do Rio de Janeiro, DIPEQ-JBRJ, Rio de Janeiro, RJ 22460-030, Brazil; Departamento de Química Orgânica/GQO, Instituto de Química, Universidade Federal Fluminense (UFF), Niterói, RJ 24020141, Brazil; Programa de Pós-Graduação em Botânica, Escola Nacional de Botânica Tropical, Jardim Botânico do Rio de Janeiro, Rio de Janeiro, RJ 22460-036, Brazil; Instituto de Pesquisas Jardim Botânico do Rio de Janeiro, DIPEQ-JBRJ, Rio de Janeiro, RJ 22460-030, Brazil; Departamento de Produtos Naturais e Alimentos, Faculdade de Farmácia, Centro de Ciências da Saúde, Universidade Federal do Rio de Janeiro (UFRJ), Rio de Janeiro, RJ 21941-902, Brazil; Instituto de Química, Centro de Tecnologia, Universidade Federal do Rio de Janeiro (UFRJ), Rio de Janeiro, RJ 22945970, Brazil; Embrapa Agroindústria de Alimentos, Rio de Janeiro, RJ 23020-470, Brazil; Programa de Pós-Graduação em Botânica, Escola Nacional de Botânica Tropical, Jardim Botânico do Rio de Janeiro, Rio de Janeiro, RJ 22460-036, Brazil; Instituto de Pesquisas Jardim Botânico do Rio de Janeiro, DIPEQ-JBRJ, Rio de Janeiro, RJ 22460-030, Brazil; Programa de Pós-Graduação em Botânica, Escola Nacional de Botânica Tropical, Jardim Botânico do Rio de Janeiro, Rio de Janeiro, RJ 22460-036, Brazil; Departamento de Produtos Naturais e Alimentos, Faculdade de Farmácia, Centro de Ciências da Saúde, Universidade Federal do Rio de Janeiro (UFRJ), Rio de Janeiro, RJ 21941-902, Brazil; Programa de Pós-Graduação em Botânica, Escola Nacional de Botânica Tropical, Jardim Botânico do Rio de Janeiro, Rio de Janeiro, RJ 22460-036, Brazil; Instituto de Pesquisas Jardim Botânico do Rio de Janeiro, DIPEQ-JBRJ, Rio de Janeiro, RJ 22460-030, Brazil

**Keywords:** Buzz pollination, division of labour, floral colour, floral volatiles, heteranthery, Leguminosae, pollen-only flowers

## Abstract

The division of labour hypothesis between stamens has explained the evolution of divergent functions between dimorphic stamens in the same flower. However, little is known about whether the distinct type of stamens differs in attractiveness to pollinators. Therefore, we investigate whether the two types of stamens commonly found in *Swartzia* have different visual and olfactory attractants. We performed observations of anthesis dynamics, registration and collection of floral visitors, measurements of reflectance of floral parts and chemical analysis of the volatile organic compounds of the floral parts of two species, *S. flaemingii* and *S. simplex*. Both species have two distinct sets of stamens: one with smaller and abundant stamens in the centre of the flower and the other with fewer but larger abaxial stamens. The sets differ in UV reflectance (only *S. simplex*) and exhibit a distinct chromatic contrast. Concerning olfactory attractiveness, aliphatic compounds make up most of the odour of the two species, both whole flowers and most of their floral organs. On the other hand, only *S. simplex* presented apocarotenoids (as ionones) and benzenoids. Furthermore, there are differences in the proportion of volatiles emitted by the stamen in both cases, as the high proportion of sesquiterpenes among the smaller stamens compared to the larger ones. In conclusion, the two types of stamens found in *S. flaemingii* and *S. simplex* show a distinct attractiveness. In addition, our data have demonstrated diverse ways of differential attractiveness both between distinct stamens set per flower and between the two species from the same pollen flowers genus.

## Teaser

The stamens of the same flower can differ in how they attract pollinators. The study by Basso-Alves *et al.*showed that the larger and smaller stamens in the genus *Swartzia*have different visual and olfactory signals. More surprising is that it was possible to observe that the strategies for attracting pollinators diverge between the two species studied. These differences include various types of signals, such as a visual pattern or a substance responsible for the floral odour, present in one species but absent in the other.

## Introduction

Flowers that depend on animals for their pollination use different signals to attract them. Depending on the cognitive apparatus of these animals, they can perceive floral signals and trigger appropriate behaviours for successful pollination (Koski 2020). The steps required for these animals include approximation, contact and interaction with the flower. The animal’s search behaviour for the floral resource allows the release of pollen grains and their touch with a receptive stigma (Russell *et al.* 2018). Therefore, the floral signals (i) must be attractive to the pollinator, (ii) must signal the location and type of resource within the flower and (iii) must stimulate appropriate behaviours to release the pollen grains. The most important of these floral signals are visual (e.g. colour and shape) (van der Kooi *et al.* 2019) and olfactory (fragrances) (Raguso 2008), especially in the case of pollination by bees (Kunze and Gumbert 2001; Bauer *et al.* 2017; Rowe *et al.* 2020). Visual signs can encompass multiple aspects of the flower and its parts, such as its shape (Giurfa *et al.* 1999; Howard *et al.* 2019), size (Essenberg *et al.* 2019), colour (including hue, saturation, and brightness) (van der Kooi *et al.* 2019) and colour patterns (Dafni and Kevan 1996; Leonard and Papaj 2011), in addition to contrast with the surroundings (van der Kooi *et al.* 2019). On the other hand, the volatile organic compounds (VOCs) of the flower provide the olfactory signals. Volatile organic compounds constitute floral fragrances that can vary in chemical composition (Knudsen and Gershenzon 2020) and emission patterns (Raguso and Weiss 2015). These VOCs can also vary spatially, generating spatial patterns of floral odour emissions (Dobson *et al.* 1996; Solís-Montero *et al.* 2018; García *et al.* 2021), which can be detected by pollinators (Raguso 2009). Spatial variation consists of either qualitative composition according to the emitting organ (i.e. specific VOCs of a given structure), or quantitative composition (in the proportion of emitted compounds) (García *et al.* 2021).

Therefore, different floral organs can also be expected to differ in attractiveness. Petals are considered to play a role in attracting pollinators, while carpels rarely display this attractive function (Teixeira *et al.* 2014). However, a question arises regarding the attractiveness of organs of the same whorl, as in the case of heteromorphic stamens. Several species of angiosperms have stamens of at least two types in their androecium (Endress 1994; Vallejo-Marín *et al.* 2010). A recurrent hypothesis to explain this dimorphism is the ‘division of labour’ hypothesis, in which one type of stamen offers pollen grains as a food resource for the pollinator, while the other set of stamens produces pollen grains destined mainly for plant fertilization (Müller 1881, 1883; Luo *et al.* 2008; Vallejo-Marín *et al.* 2009). Although this explanation does not apply to all cases (e.g. Peach and Mazer 2019; Telles *et al.* 2020; Dellinger *et al.* 2021), it is a hypothesis that has wide support in the literature (e.g. Paulino *et al.* 2016; Solís-Montero *et al.* 2018; Velloso *et al.* 2018; Saab *et al.* 2021; Valadão-Mendes *et al.* 2022). Thus, based on the hypothesis of ‘division of labour’ between the stamens, it could be assumed that there is a difference in the attractiveness between the distinct stamens, in which some stamens would be more attractive (*feeding stamens*) and others would be more cryptic (*pollination stamens*) (e.g. Solís-Montero *et al.* 2018; Velloso *et al.* 2018; Saab *et al.* 2021).

**Figure 1.**
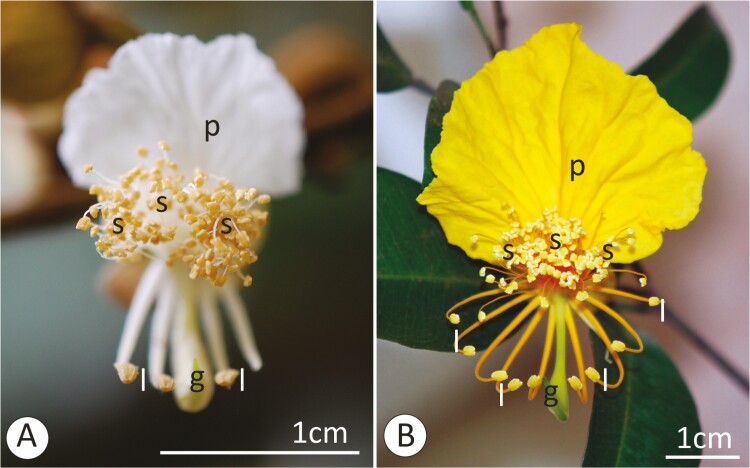
Flowers of *Swartzia flaemingii* (A) and *Swartzia simplex* var. *grandiflora* (B). Symbols: g = stigma; l = larger stamens; p = petal; s = smaller stamens.

A fascinating model for studies of stamen dimorphism is *Swartzia* (Fabaceae). Flowers of *Swartzia* species are polystemonous and usually have many (smaller) stamens in the centre of the flower and few (larger) stamens facing the abaxial region, flanking the single carpel (see Fig. 1). Thus, the heteromorphic *Swartzia* stamens differ in size, position and number (Cowan 1968). *Swartzia* flowers do not offer any resource other than pollen (Endress 1994) and are therefore called ‘pollen flowers’ (*sensu*  Vogel 1978). Stamens in the subfamily Papilionoideae are rarely heteromorphic (see Paulino *et al.* 2016). Furthermore, *Swartzia* differs from the other genera of the Papilionoideae by not having the typical papilionaceous flowers (Tucker 2003; Paulino *et al.* 2013). *Swartzia* is a Neotropical group with more than 190 species, grouped into 15 monophyletic sections (Torke and Mansano 2009). The morphological differences between the stamens are due to their differential development (Tucker 2003; Paulino *et al.* 2013). Whether such structural differences in *Swartzia* provide differences in the attractiveness of these stamens remains unclear.

The bees that pollinate *Swartzia* flowers are capable of vibrating the stamens (Lopes and Machado 1996; Moço and Pinheiro 1999; Pinheiro *et al.* 2018). The arrangement of the stamens in *Swartzia* and the behaviour of these bees for pollen collection suggest that the smaller stamens are associated with feeding the pollinator while the larger stamens are linked to the reproductive role of pollination. The differential attractiveness of stamen whorls has until now been poorly studied. However, there are differences in visual perceptibility between stamens (Velloso *et al.* 2018; Telles *et al.* 2020; Saab *et al.* 2021). In the same way, variations in the pattern of volatiles emitted by stamens were detected in some cases (Solís-Montero *et al.* 2018). Nonetheless, there is still a lack of research that combines both approaches to test the differential attractiveness for each stamen type of the same flower. Thus, the aim of this study was to investigate whether there are differences between visual and olfactory signals, leading to a differential attractiveness in the *Swartzia* dimorphic stamens. Furthermore, we intend to compare the homogeneity of floral attractants between two species of the same genus.

## Materials and Methods

### Study system

The two species of *Swartzia* selected for this study belong to different sections (Torke and Mansano 2009) and occur naturally in the Brazilian Atlantic Forest (BFG 2018). *Swartzia flaemingii* (Fig. 1A), section *Acutifoliae* (Torke and Mansano 2009), is preferentially found in areas of Montana Dense Ombrophilous Forest (Silva 2010); while *Swartzia simplex* var. *grandiflora* (Fig. 1B) [hereafter *Swartzia simplex*], section *Possira* (Torke and Mansano 2009), is found in areas of Lowland Dense Ombrophilous Forest and coastal shrubland and forest (*Restinga*) (Silva 2010). Specifically for this study, we monitored and collected materials from individuals of *S. flaemingii* [RB 362170, RBv 2451] and *S. simplex* [RB 799144, RBv 2580] cultivated in the Arboretum of the Rio de Janeiro Botanical Garden. We followed two successive flowering periods. *Swartzia flaemingii* flowered from late January to March (2018–19) and *S. simplex* from October to December (2017–19).

### Floral biology

Focal observations were made on days with clear skies, with the help of scaffolding installed next to the plants. Images were captured with a Nikon DSLR D7200 Digital Camera. Pinheiro *et al.* (2018) studied the floral visitors of both species (however, *S. flaemingii* was referred to as ‘*Swartzia oblata*’), while our observations were focused on the pollen collection behaviour and interaction of the animals with the floral parts. Visitors were grouped according to their pollen gathering behaviour following the terminology proposed by Portman *et al.* (2019). These behaviours include: scraping with the extremities, buzzing, rubbing with the body and/or scopae, rubbing with the face, tapping and rasping. Several bees were captured for identification by a specialist (see Acknowledgements).

The opening dynamics of the anthers were complemented with an Olympus SZ61 stereomicroscope with an Olympus SC30 digital camera.

### The reflectance of the floral parts

To test for visual crypsis of the larger stamens, as opposed to the smaller stamens that are more likely to act as attractants (Vallejo-Marín *et al.* 2009; Xiong *et al.* 2019), we compare the mean contrasts of both kinds of stamens in the bee visual system, using leaves and corolla as the background.

Leaf and flower samples were collected for spectral analysis. We took at least 15 reflectance measurements for each of the following structures: petals, smaller stamens and larger stamens, besides five leaf reflectance measurements. For structures large enough, i.e. petals, we also investigated a change in reflectance along the surface as one moves toward the centre of the flower, as observed for some species that present a ‘bull’s eye’ reflectance pattern (Lunau *et al.* 2021), with a UV-reflecting periphery and a UV-absorbing centre. Since we detected no intra-structure variability in reflectance, we averaged all measurements taken for each structure. The reflectance of the samples was measured using a portable spectrometer (Ocean Optics USB 4000; Ocean Optics Inc., Dunedin, FL, USA) at an angle of 45°, and using barium sulfate (BaSO_4_) as the white standard and a black chamber as the black standard (Lunau *et al.* 2011; Bergamo *et al.* 2016). Structures smaller than 10 mm, like stamens, were arranged like fish scales to expose a single colour to the spectrometer (Chittka and Menzel 1992).

The spectral data were then used to assess petal and stamen conspicuousness according to the visual system of *Bombus terrestris*, an important model bee species for which the sensibility data were available in the literature (Peitsch *et al.* 1992). The utility of this well-studied visual system is that spectral sensitivity has been shown to be a conservative trait in Hymenoptera (Peitsch *et al.* 1992). The chromatic and achromatic contrasts against the background (henceforth CCB and ACB) were used to determine conspicuousness to bees (Coimbra *et al.* 2020).

CCB and ACB were computed according to the bee chromaticity diagram of Chittka (1992) and the daylight illuminant function D65 using the ‘*vismodel*’ function of the ‘pavo’ package (Maia *et al.* 2019) in R (R Core Team 2018). The mean contrast of the two types of stamens was compared using ANOVA tests, for both petal and leaf backgrounds. This is because the corolla shape of the studied species allows for both scenarios depending on the visual angle. The mean corolla contrast between the two species was also compared.

### Floral odour

#### Flower collection.

Around 30 flowers were collected early in the morning, when the flowers open, to analyse the VOCs. After collection, the flowers were kept at a low temperature (−20 °C) until chromatographic analysis. The floral odour was obtained for the whole flowers and each organ separately (petals, larger stamens, smaller stamens and carpels) by solid-phase microextraction (SPME) and analysed by gas chromatography and mass spectrometry (GC-MS).

#### Solid-phase microextraction.

The floral odour of the investigated species was collected using the SPME technique. Before the first use, the collection device containing a divinylbenzene/carboxene/polydimethylsiloxane fibre (DVB/CAR/PDMS; 50/30 µm; Supelco Inc., Bellefonte, PA, USA) was conditioned for 60 min at 270 °C in the chromatographic injector, according to the manufacturer’s instructions. Also, the fibre was thermally cleaned in the chromatographic injector at 270 °C, for 10 min, between the sampling replicates immediately before each extraction procedure.

The extraction of volatiles from whole flowers used three flowers of each species that had been previously collected. In order to sample the volatiles of each floral organ, 300–500 mg were used for each sampling, according to the mass available.

Each type of floral sample was placed in a sealed flask, which, in turn, was subjected to a fixed temperature of 40 °C for 30 min. This time was considered to be sufficient to achieve partition equilibrium. After the equilibrium time, the SPME fibre was exposed to the flower’s headspace for 20 min. After the extraction time, the fibre was collected and, immediately afterward, introduced into the chromatographic injector for 3 min for desorption and analysis of the extracted analytes.

#### Qualitative gas chromatography analysis.

The analysis of the profile of volatiles extracted by the SPME fibre was performed on an Agilent 7890A gas chromatograph equipped with a 5975C mass spectrometer. An HP-5MS capillary column (5 % phenyl-methylpolisyloxane; 30 m × 0.25 mm × 0.25 μm) of fused silica was used. Helium was the carrier gas with a flow rate of 1.0 mL min^−1^. The mass detector was operated in electronic ionization mode (70 eV), at 3.15 scans per second, with a mass range from 40 to 450 μ. The transfer line was maintained at 260 °C, the ion source at 230 °C and the analyser at 150 °C. The chromatographic oven was programmed from 40 °C (5 min) to 240 °C at 3 °C min^−1^. The SPME fibre was exposed in an injector at 250 °C in the splitless mode. The chromatographic injector used a 0.75-mm internal diameter liner, suitable for analyses with SPME.

#### Identification of volatiles.

The identification of volatiles was performed by comparing the mass spectrum of the compounds under analysis with mass spectra data available in the literature and in the database of the Wiley Registry of Mass Spectral Data 6th ed. (McLafferty and Stauffer 1994). Simultaneously comparing the calculated linear retention indices (LRI) with those available in the literature (Adams 2007). The LRI calculation was carried out by injecting a homologous series of *n*-alkanes C_7_–C_26_ according to van Den Dool and Kratz (1963).

## Results

### Floral structure and anthesis dynamics

**Figure 2.**
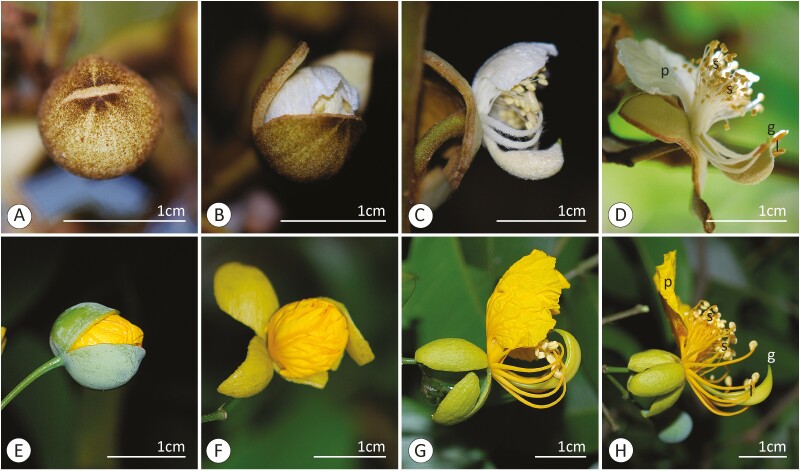
Dynamics of anthesis of *Swartzia flaemingii* (A–D) and *Swartzia simplex* (E–H). (A, E) Rupture of the calyptrate calyx. (B, F) Opening of the broken calyx. (C, G) Distension of petals, stamens and carpel. (D, H) Flower with fully unfolded organs. Symbols: g = stigma; l = larger stamens; p = petal; s = smaller stamens.

The structure and anthesis dynamics of the *S. flaemingii* and *S. simplex* flowers were very similar, so they will be described together. However, these flowers differ in size and colour (Figs 1 and 2), with white petals and filaments in *S. flaemingii* and yellow in *S. simplex*. Furthermore, the flowers of *S. simplex* were found in axillary inflorescences, while the inflorescences of *S. flaemingii* are located on leafless branches **[see**  **Supporting Information—Fig. S1****]**.

A united, calyptrate calyx enclosed the floral buds of both species. The opening of the calyx began around 1800 h, with the formation of distal cracks on the calyptra (Fig. 2A). These slits extended towards the floral receptacle (Fig. 2B and E). As a result, the calyx lobes slit and other floral organs were exposed (Fig. 2C and F). The complete release of the calyx fragments occurred at 0000 h. At this stage the calyx has become reflexed. Thus, the single petal lining of the bud was exposed and it distended (Fig. 2C and G), becoming erect and flat (Fig. 2D and H). At the same time, the carpel, and the larger stamens, which were curved, unfold in the abaxial direction (Fig. 2C and G). Filaments of the smaller stamens also unrolled, although not all these stamens were completely erect due to their thickness. Thus, the smaller stamens were more or less parallel to the axis of the floral pedicel, while the petal was perpendicular in the adaxial region, and the base of the larger stamens and carpel were perpendicular in the abaxial region. This structure could be seen in the flowers before 0600 h and culminated in the strongly monosymmetric appearance of the flowers (Figs 1 and 2). The opening of the anther, of both types of stamens, has begun with the stamens still bent over the bud. However, the opening may not be complete in most of the smaller stamens at this time. The anthers of both species and all types of stamens showed longitudinal dehiscence **[see**  **Supporting Information—Fig. S2****]**. The pollen grains had a powdery and whitish appearance.

### Floral visitors

**Figure 3.**
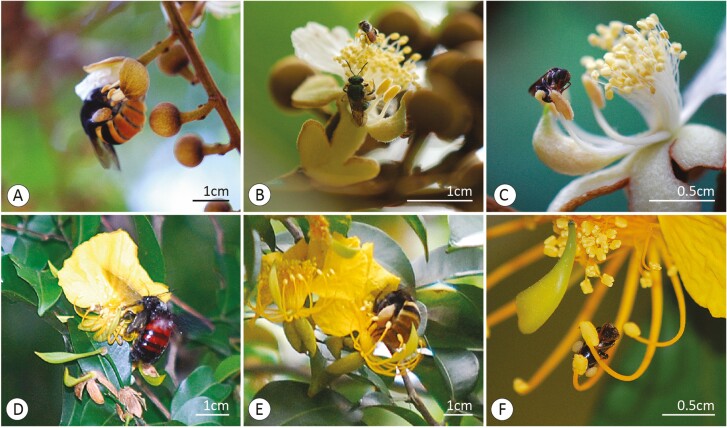
Bees that visit *Swartzia* flowers behave in two main ways: large bees (‘legitimate visitors’) vibrate the stamens to collect pollen (A, D, E) and smaller bees (‘illegitimate visitors’) collect pollen directly from the anthers (B, C, F). Flowers of *Swartzia flamingii* visited by species of *Epicharis* (A), *Tetragonisca* (above) and Halictideae (below) (B) and *Paratrigona subnuda* (C). Flowers of *S. simplex* visited by species of *Xylocopa* (D), *Eulaema* (E) and *Nannotrigona* (F).

Bees that collect pollen by vibrating the stamens behaved basically the same way for both species of *Swartzia*. The bees made a frontal approach to the flower, landed on the smaller stamens, which were then vibrated to release their pollen. This vibration was transmitted to the entire flower, including the larger stamens. In this way, the pollen dispersed like a small cloud over the bee’s body (sternotribic/nototribic deposition). The pollen from the smaller stamens was collected mainly in the ventral region of the bee. The backs of these bees also came in contact with the anthers of the larger stamens, in addition to the stigma. Then, the bee releases the smaller stamens and moves on to another flower. After a few visits, the bee performs its grooming, brushing the pollen deposited on its body and collecting it in its corbiculae. This group of female bees included ‘large’ bees such as species of *Epicharis*, *Eulaema* and *Xylocopa* (Fig. 3). The other group of visitors included smaller female bees such as species of stingless *Melipona*, *Nannotrigona* and *Oxytrigona* (Fig. 3). In this later group, other types of pollen collection behaviours were found, e.g. scraping with the extremities or rubbing with the face (*sensu*  Portman *et al.*, 2019).

### Visual signals

**Figure 4.**
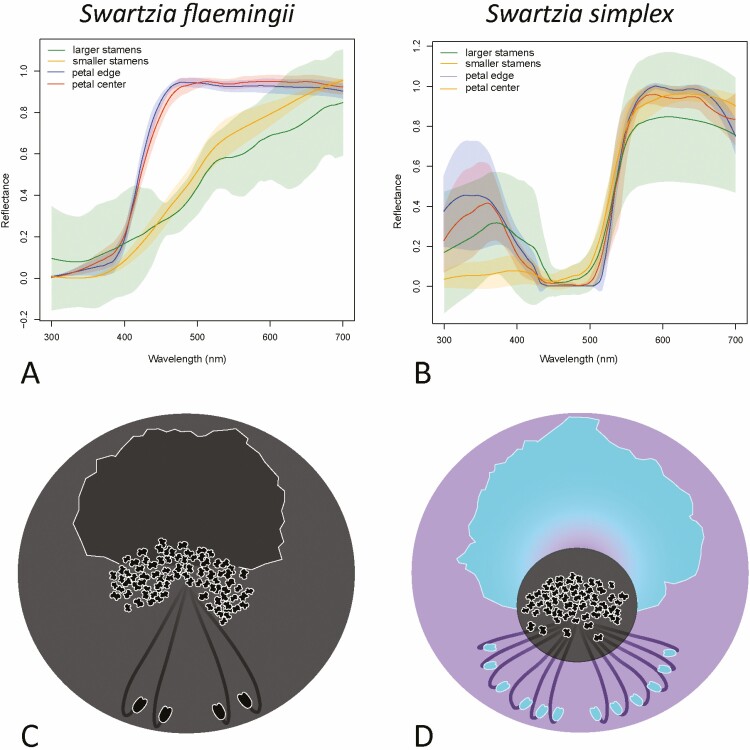
Reflectance spectra of the floral parts in *Swartzia flaemingii* (A) and *Swartzia simplex* (B). UV absorption/reflection pattern in schematic flowers of *S. flaemingii* (C) and *S. simplex* (D). Both types of stamens and the petal absorb UV, generating no UV contrasting pattern between these organs in *S. flaemingii* (C). In contrast, *S. simplex* has UV-absorbing smaller stamens, but UV-reflecting larger stamens and petals.

*Swartzia flaemingii* flowers had white petals—UV-absorbing, and creamy stamens—also UV-absorbing (Fig. 4A). *Swartzia simplex*, however, presented a UV-reflecting yellow petal and larger stamens, but UV-absorbing yellow smaller stamens (Fig. 4B). Thus, for *S. simplex*, the two types of stamens differed markedly in UV reflection (Fig. 4D), while for *S. flaemingii* no clear difference was visible (Fig. 4C). Since larger stamens and petals were displayed in an outer position regarding the central position of the smaller stamens, this could create a bull’s eye reflectance pattern (*sensu*  Lunau *et al.* 2021) when the flower was viewed by an approaching bee: an outer UV-reflecting periphery and an inner UV-absorbing centre (Fig. 4D).

We found higher chromatic (CCB) and achromatic contrasts (ACB) for the petals of *S. flaemingii* than for *S. simplex*  **[see**  **Supporting Information—Fig. S3****]**. This means that *S. flaemingii* displayed flowers that were more conspicuous against a leaf background and hence more easily detectable to bumblebees than *S. simplex*.

**Figure 5.**
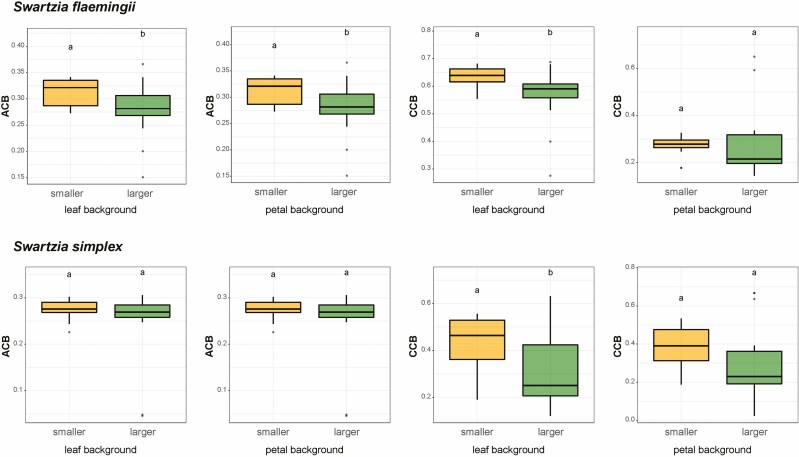
Mean chromatic (CCB) and achromatic (ACB) contrast differences between smaller and larger of stamens of *Swartzia flaemingii* and *Swartzia simplex* against a leaf and a petal background. Contrasts are given in hexagon units according to the visual system of *Bombus terrestris*.

*Swartzia flaemingii* stamens had less pronounced differences from each other in the hexagon model than those of *S. simplex*  **[see**  **Supporting Information—Fig. S4****]**. Furthermore, the stamens of both *S. flaemingii* and *S. simplex* differed in CCB against a leaf background (*P* = 0.01 and *P* = 0.03, respectively, Fig. 5), with higher chromatic contrast for the smaller stamens than for the larger stamens, as seen from their relative distances from the hexagon centre **[see**  **Supporting Information—Fig. S4]**. The stamens of *S. flaemingii* also differed in ACB (*P* = 0.02, Fig. 5), following a similar pattern: larger stamens contrasted less than smaller stamens against both petal and leaf backgrounds.

### Chemical signals

#### Floral volatiles

**Figure 6.**
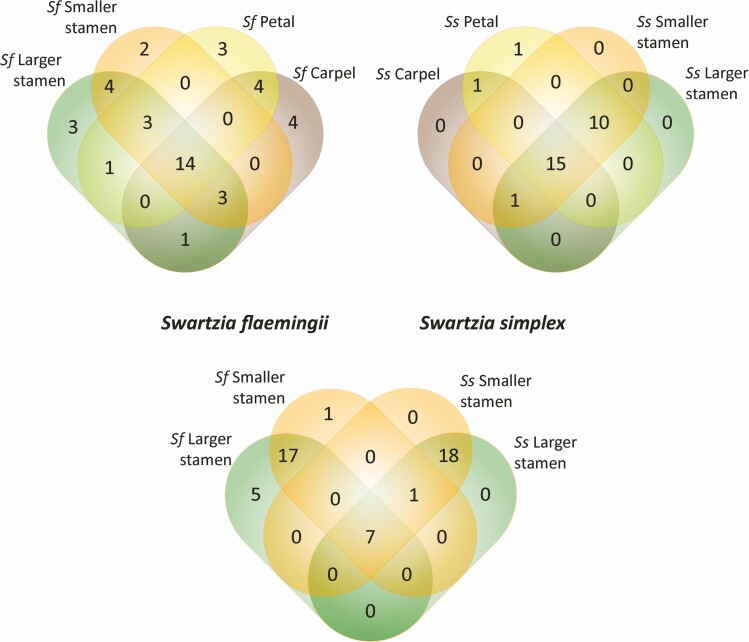
There is qualitative overlap between all the VOCs of the *Swartzia simplex* stamens, while there are some unique VOCs of each stamen type in *S. flaemingii*. Venn diagrams show the number of compounds per flower organ in each species (upper panel) and the comparison between the number of compounds emitted by each type of stamen in *S. flaemingii* and *S. simplex* (bottom panel).

The VOCs of both species were composed of compounds from the same biosynthetic classes (Table 1), although the chromatographic profiles were different **[see**  **Supporting Information—**Appendix **S5****]**. Such differences are reflected in the number and chemical identity of compounds found in each species, and their relative proportions (Table 1; Fig. 6; **see**  **Supporting Information—**Appendix **S5**). The GC-MS protocol used was qualitative, so are the nature of the results. Therefore, no quantitative data can be directly compared. Regarding relative proportions, only a rough approximative inference can be performed (Table 1).

**Table 1.**
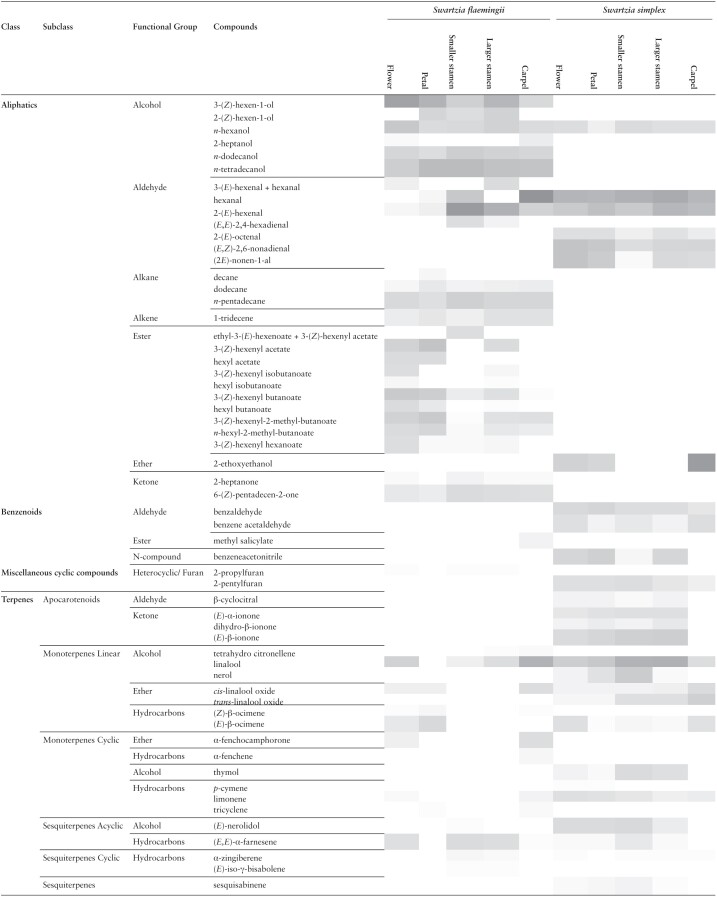
Synopsis of the floral volatiles of *Swartzia flaemingii* and *Swartzia simplex*, according to the classification of functional groups by Knudsen *et al.* (2006). The relative abundance of each substance in the total floral odour composition is indicated by a gradient, in which gray indicates greater intensity and white less intensity.

The floral VOC of *S. flaemingii* had more compounds (*N* = 42) than *S. simplex* (*N* = 28) (Fig. 6). However, the floral odour in *S. simplex* was more intense than in *S. flaemingii*. The two flower species had 10 compounds in common (hexanal; 2-(*E*)-hexenal; *n*-hexanol; linalool; (*Z*)-linalool oxide; (*E*)-nerolidol; limonene; α-zingiberene; (*E,E*)-α-farnesene; (*E*)-β-ocimene). Aliphatic compounds predominated in the floral odour of both species. However, among these, *S. flaemingii* had more types of alcohols and *S. simplex* more types of aldehydes (carbon chains of 6–9 carbons, as opposed to C6 aldehydes from *S. flaemingii*). Furthermore, *S. flaemingii* had alkanes, alkenes, ketones, esters and other functional groups not found among the aliphatic compounds of *S. simplex*. On the other hand, only *S. simplex* presented apocarotenoids (as ionones) and benzenoids.

There are two distinct situations from a qualitative point of view in relation to the volatiles emitted by stamens (Fig. 6; Table 1; **see**  **Supporting Information—**Appendix **S5**). The same 26 compounds were found in the smaller and larger stamens of *S. simplex*. In *S. flaemingii*, on the other hand, 26 compounds were found in the smaller stamens and 29 in the larger ones, of which 22 are common to both stamen types. However, there are five compounds that were not common to the two stamen types, nor were they found in any other floral organ. Ethyl-3-(*E*)-hexenoate and (*E*)-nerolidol were only found in the smaller stamens and hexyl isobutanoate; 3-(*E*)-hexenal and 3-(*Z*)-hexenyl isobutanoate in the larger stamens. In *S. simplex*, terpenic alcohols such as nerol and (*E*)-nerolidol produced more intense peaks in the smaller stamens compared to the larger ones. Some aliphatic compounds seem to be more abundant in the larger stamens than smaller ones, such as 2-(*Z*)-hexen-1-ol, 3-(*Z*)-hexen-1-ol and *n*-hexanol in *S. flaemingii* or 2-(*E*)-hexanal in *S. simplex*. Linalool was present in larger proportions in the larger stamens of the two species.

The only pattern observed in relation to the volatiles of both species was the high proportion of sesquiterpenes among the smaller stamens compared to the larger ones. In other groups of compounds, the differences can be the inverse between species or stamens. For example, aldehydes were more abundant in the smaller stamens of *S. flaemingii* than the larger stamens of *S. simplex*, while alcohols were more abundant in the larger stamens of *S. flaemingii* than the smaller stamens of *S. simplex*.

## Discussion

The heteromorphic stamens of the two studied species of *Swartzia* differ in important functional aspects. In addition to the attractiveness differing between the two sets of stamens, our study also reveals how an apparently homogeneous system of pollen flowers of the same genus can differ in the strategies used to attract pollinators. In both species the larger stamens appear cryptic in relation to the smaller ones, especially in relation to the achromatic contrast and relative abundance of some VOCs attractive to bees, such as sesquiterpenes. However, the greater attractiveness of the smaller stamens is also due to intrafloral patterns of UV reflectance and presence of VOCs more specialized in *S. simplex*.

### The floral biology of *Swartzia* species

Some aspects of the interaction between the flowers of *S. flaemingii* and *S. simplex* with the floral visitors seem to be recurrent in the other species of *Swartzia* (Lopes and Machado 1996; Moço and Pinheiro 1999; Pinheiro *et al.* 2018). One of them is that bees that collect pollen by vibrating the stamens always grab the smaller stamens. Another aspect is that both types of stamens release their pollen grains during the same vibration event. However, the pollen deposition site differs in relation to the bee body (Pinheiro *et al.* 2018). The pollen released by the smaller stamens is preferentially deposited on the ventral region of the bee (sternotribic deposition). While the pollen from the larger stamens reaches the dorsal abdomen (nototribic deposition). Bees easily remove the pollen available in its ventral region during grooming, thus accessing it as food for larvae. In contrast, pollen that reaches the dorsal abdomen is more difficult to remove. This latter observation is crucial for recognizing ‘safe sites’ for plant reproduction (Koch *et al.* 2017; Tong and Huang 2018).

The *Swartzia* species in general, despite very similar anthesis dynamics, present considerably different pollinator guilds (Lopes and Machado 1996; Moço and Pinheiro 1999; Pinheiro *et al.* 2018). The flowers of *Swartzia flaemngii* and *S. simplex* are visited by different groups of bees, such as carpenters, bumblebees, oil bees and stingless bees. This denotes that both *Swartzia* species employ attractive signals to a wide range of bees. But the pollinator guild of *S. simplex* is smaller (less diverse) than that of *S. flaemingii*, although most bee species that visit one species of *Swartzia* also visit the other one (Pinheiro *et al.* 2018). The main reason for the difference in the pollinator guild seems to be the difference in the size of floral organs. The stamens and carpels of *S. simplex* are larger than those of *S. flaemingii*, being hard to the smaller bees to have body contact with the stigma when collecting pollen. The separation distance between stigma and anthers seems to impose a threshold of minimum size on the bee’s body so that it can be effective in the role of pollinator (see Mesquita-Neto *et al.* 2021). Thus, Pinheiro *et al.* (2018) consider that only large bees (>20 mm, e.g. some *Xylocopa*) would be pollinators of *S. simplex*, while *S. flaemingii* could be pollinated by medium bees (>12–20 mm, e.g. *Bombus*, *Epicharis*), in addition to large bees (e.g. *Eulaema*, *Xylocopa*). However, large bees of the genus *Eulaema*, despite being found in the flowers of *S. simplex*, were not considered pollinators due to their low frequency of visit (Pinheiro *et al.* 2018). The minimum bee size threshold also seems important for other *Swartzia* species, such as *Swartzia apetala* (sect. *Swartzia*), pollinated by *Centris* and *Xylocopa* (Moço and Pinheiro 1999) and *Swartzia pickelii* (sect. *Acutifoliae*), pollinated by *Eulaema* (Lopes and Machado 1996).

We hypothesize that smaller stamens have greater mechanical cohesion than larger stamens. Two main factors provide this cohesion. One of them is that the smaller stamens are formed from the same meristematic mass, similar to a ring (Tucker 2003; Paulino *et al.* 2013), which promotes a deep structural bond between them. The other factor is that the filaments of the smaller stamens are not fully extended during anthesis, which results in their having a more or less intertwined arrangement. This cohesion drastically affects the ability of the anthers to release pollen grains. An example is *Huberia bradeana* (Melastomataceae), which has stamens intertwined by elongated and sinuous appendages of the connective, in which the experimental removal of these appendages leads to a retention of pollen grains in the vibrated anther (Bochorny *et al.* 2021). Thus, the intertwining of the smaller stamens allows the vibrations of the bee to be transmitted to the other stamens of *Swartzia* easily and even the flower as a whole when the bee grabs a few stamens to vibrate the anthers. Few studies have been able to demonstrate how the flower transmits this vibration by the bee (Vallejo-Marín 2019; Brito *et al.* 2020). However, this seems to be a key component in understanding the evolution of pollination by vibration (Brito *et al.* 2020), in addition to aspects currently being investigated such as the ability of bees to vibrate (see Vallejo-Marín 2022). The organization of the floral parts, such as the interweaving of the smaller stamens in *Swartzia*, may be a case in point.

The pollen collection behaviour by vibration is closely related to the restriction of pollen release by the anther (Buchmann 1983; Cardinal *et al.* 2018). Poricidal anthers represent the best-known limitation for vibration-pollinated flowers (Buchmann 1983; Endress 1994; Russell *et al.* 2017; Cardinal *et al.* 2018). However, this type of dehiscence is not mandatory for buzz pollination (Buchmann 1985), as noted in *Swartzia*. Modified configurations of longitudinal dehiscence or small basal and apical openings of the dehiscence line, such as a shortening of the anther slit, can be very close to the poricidal anthers (Endress 1996; Saab *et al.* 2021). In addition to restricting the release of pollen, other strategies can favour the saving of pollen by the flower in *Swartzia*. One of them is the cryptic pollen condition (Xiong *et al.* 2019). When the pollen has less contrast than the anther, this appears to prevent excessive pollen removal by legitimate visitors and elude pollen thieves (Xiong *et al.* 2019). Such alternatives could compensate for the open floral construction of *Swartzia*, which does not present barriers for visitors to access the anthers, the only source of floral resource.

### The visual attractiveness of the flowers

A conspicuous visual difference between the flowers of the two *Swartzia* species, besides size, is the colours under human perception—white in *S. flaemingii* and yellow in *S. simplex*. These colours are recurrent in bee-pollination systems and do not show, *per se*, a greater or lesser preference concerning the visiting behaviour of the bees. The colour of the single petal of *Swartzia* flowers varies by species (Cowan 1968; Torke and Mansano 2009; BFG 2018). Almost half of the *Swartzia* species (at least 55) have has white petals (sect. *Acutifoliae*, *Benthamianae*, *Glabriplantae*, *Orthostylae* and *Unifoliolatae*), while the other half (at least 71) have yellow petals (sect. *Circumnodae*, *Paucistaminae*, *Pittierianae*, *Possira*, *Recurvae* and *Terminales*), and some species (circa 27) are apetalous (mainly from the sect. *Swartzia* and some from the sect. *Terminales*) (Torke and Mansano 2009). Because of the polytomy in the phylogeny of *Swartzia* (see Torke and Mansano 2009), it is not possible to infer which one of the early-branching lineages of *Swartzia* (clade 1: *Glabriplantae*, clade 2 *Benthamianae* + *Orthostylae* and clade 3 with the remaining sections), it is not possible to infer whether ‘yellow petals’ represent independent acquisition events within the genus; however, the petal colour seems to be conservative within each section (= each subclade).

We found no UV gradients or any intra-structure colour variations that could act as floral guides in the petals of either species. However, for S. *simplex* flowers, we realized a general UV pattern similar to the ‘bull’s eye’ pattern, as seen for some bee-pollinated flowers (e.g. Lunau *et al.* 2021; Tunes *et al.* 2021). This pattern refers to flowers displaying a UV-reflecting periphery but a UV-absorbing centre. In *S. simplex*, this reflectance pattern consists of a UV-reflecting petal and larger stamens, which partially surround the UV-absorbing smaller stamens in the centre of the flower (see Fig. 4D). Thus, the increase in the number of larger stamens may be associated with the configuration of this visual pattern in *S. simplex*. The number of larger stamens is a highly variable character in *Swartzia* (Cowan 1968; Torke and Mansano 2009; BFG 2018). Most species have a maximum of four larger stamens (as in the case of *S. flaemingii*), but cases with more than 10 larger stamens are usual among species in the sections *Multistaminae*, *Possira* (including *S. simplex*), some *Pittierianae*, *Recurvae* and *Terminales*. Interestingly, flowers with many larger stamens are always yellow, while species without petals always have few larger stamens, usually two (except *Paucistaminae*, which has a yellow petal and few larger stamens). Furthermore, a UV-absorbing yellow centre could increase the attractiveness of smaller stamens in *S. simplex*, as this colour category is closely related to pollen mimicry in bee-pollinated flowers (Lunau 2000). Yellow flowers often exhibit some UV reflectance pattern (Lunau *et al.* 2021). On the other hand, UV-absorbing white flowers, as found in *S. flaemingii*, compose the most frequent colour category in bee-pollinated systems, which also the most conspicuous colour category to bees (Coimbra *et al.* 2020).

The main difference in visual attractiveness between *Swartzia* stamens concerns the achromatic contrast. Although the measured achromatic contrast against a leaf background of *S. flaemingii* petals is greater than that of *S. simplex* petals, they may not differ much in attractiveness under natural conditions (Koski 2020). This is because *S. flaemingii* inflorescences display flowers covered by leaves, unlike *S. simplex*, where most of the flowers are exposed in the crown, besides having much larger flowers. Shaded flowers are less visited in some systems, which would favour a selection towards more attractive signals in such environments to compensate for the degradation conditions on signal transmission between flowers and floral visitors (Koski 2020). Although chromatic contrast is usually assumed to be a noticeable signal at shorter distances when compared to achromatic contrast in bees, particularly *Apis mellifera* (Spaethe *et al.* 2001), in reality, little is known about its role for the bees considered here as legitimate visitors. Some species of stingless bees and bumblebees use chromatic and achromatic contrasts at similar distances (van der Kooi *et al.* 2019). Thus, in addition to the integration of multimodal stimuli, the communication effectiveness must consider the interference of the floral context during signal emission.

### The olfactory attractiveness of flowers

The compounds that make up the floral volatiles of *Swartzia* are ubiquitous in angiosperms in general. Thus, the chemical profile of the two *Swartzia* sampled here suggests a broad-spectrum chemical repertoire to attract bees (Dobson 2006). The specializations found here relate more to the role of each organ in attracting the pollinator. Chemical variations include differences in which VOCs are emitted by each organ and differences in the relative proportions of each (García *et al.* 2021).

The VOCs found in *Swartzia* flowers are formed mainly by compounds recognized as generalist attractants; that is, they are emitted by a wide range of plants pollinated by different groups of pollinators, including bees, butterflies and flies. This is the case, for example, of the monoterpenoids linalool and (*E*)-β-ocimene—found, respectively, in the floral odour of 70 % and 71 % of the plant species already studied (Filella *et al.* 2013; Knudsen and Gershenzon 2020). In addition, the large number of compounds emitted by *S. flaemingii* and *S. simplex* flowers, as well as the presence of compounds from different biosynthetic pathways, reinforce the idea that such volatile profiles together with other floral characters work together to attract a wide range of floral visitors (Tollsten *et al.* 1994).

The existence of a spatial variation in the emission of VOCs, as found for *S. flaemingii* and *S. simplex*, has important consequences for their floral biology (García *et al.* 2021). Quantitative differences in volatile release between stamens can result in the intrafloral patterns that may be perceptible to the floral visitors. Some species of bumblebees, such as *B. terrestris*, are able to distinguish spatially distinct olfactory patterns (Lawson *et al.* 2018), even if a single uniform odour constitutes such patterns; that is, the odour comes from identical qualitative chemical compositions.

Different studies have shown that the electro-antennographic response of bee antennae, as well as of other insects, is dependent on the concentration of compounds; that is, the antennae have mechanisms capable of recognizing quantitative differences present in the olfactory context (Du and Millar 1999; Hieu *et al.* 2014; Wang *et al.* 2016; Chen *et al.* 2019). Thus, even in the case of *S. simplex*, which presents the same VOCs for both stamens, the olfactory perception between them must differ, given their distinct relative abundance. Some sesquiterpenoids, such as (*E,E*)-α-farnesene and (*E*)-nerolidol, which are more abundant in the smaller stamens in both species of *Swartzia*, are known to attract bees, and elicit electro-antennographic responses when subjected to analysis with gas chromatography-electroantennographic detection (GC-EAD) (Dötterl *et al.* 2005). The presence of (*E*)-nerolidol is significantly associated with pollination by large bees such as *Xylocopa* (Rabeschini *et al.* 2021).

The presence of ionones in *S. simplex* flowers, such as (*E*)-β-ionone, is a strong indication of pollination by *Xylocopa*. Although β-ionone is not a specific signal for attracting carpenter bees, as it is attractive to other types such as stingless bees and euglossini males, it has been considered a reliable marker for pollination by *Xylocopa* (Rabeschini *et al.* 2021). In this sense, *S. simplex* is more specialized in attracting large bees of this type than *S. flaemingii*, in which we do not find ionones in its floral bouquet. It is interesting to note that coincidentally ionones are observed only in *S. simplex*, since ionones and carotenoids, involved in the production of floral pigments, such as yellow pigments, have the same biosynthetic precursors (Sun *et al.* 2018).

## Conclusions

The two types of stamens, found in *S. flaemingii* and *S. simplex*, exhibit differences that imply a distinct attractiveness between them. Thus, the data obtained here support the hypothesis that the larger stamens (pollinating stamens) are cryptic compared to the smaller stamens (feeding stamens) for *Swartzia*. Furthermore, our study also reveals how species with dimorphic stamens of the same genus can use different strategies in this functional separation. This brings insights not only to the understanding of the diversification of a genus as large as *Swartzia*, but also highlights the importance of investigating different types of signals in the same system with division of labour between stamens.

## Supporting Information

The following additional information is available in the online version of this article—

Figure S1. Inflorescences of *Swartzia flaemingii* (A) and *Swartzia simplex* (B).

Figure S2. Anthers of *Swartzia*. (A) Anther of the smaller stamen of *Swartzia flaemingii*. (B) Anther of the larger stamen of *S. flaemingii*. (C) Anther of the smaller stamen of *S. simplex*. (D) Anther of the larger stamen of *S. simplex*.

Figure S3. Mean chromatic (CCB) and achromatic (ACB) contrast differences for the petals of *Swartzia flaemingii* and *Swartzia simplex* against a standard green leaf background. Contrasts are given in hexagon units according to the visual system of *Bombus terrestris*.

Figure S4. Visual modelling of stamen colour using the colour hexagon model for *Bombus terrestris*. The hexagon diagrams represent the relative excitation of each of the three bee photoreceptors (E): ultraviolet (UV), green (G) and blue (B). The points indicate how conspicuous each stamen type is according to the hexagon colour model against different backgrounds. Thus, the furthest a point is located from the centre of the hexagon, the more visible it is to bee vision, as it stands out from the leaf/petal background (represented by the centre of the hexagon). Likewise, the closer two given points are located from each other, the less distinguishable they are in bee vision.

Appendix S1. Chromatograms – *Swartzia flaemingii*.

Table S1. Constituents and relative percentage of volatile identified in whole flowers (WF) and isolated flower organs (Pet: petals; Ssta: Smaller stamens; Lsta: Larger stamens; Car: carpel) from *Swartzia flaemingii* by HS-SPME-GC-MS. 

Appendix S2. Chromatograms – *Swartzia simplex*.

Table S2. Constituents and relative percentage of volatile identified in whole flowers (WF) and isolated flower organs (Pet: petal; Ssta: Smaller stamen; Bsta: Larger stamen; Car: carpel) from *Swartzia simplex* by HR-SPME-GC/MS.

plac041_suppl_Supplementary_Material

plac041_suppl_Supplementary_Data

## Data Availability

Raw spectral data for *Swartzia* leaves and floral parts are available in Spectral data.xlsx.

## Notes

**Natural History and Conservation. Chief Editor:** F. Xavier Picó
