# **Correction to:** Heteromorphic stamens are differentially attractive in Swartzia (Fabaceae)

**DOI:** 10.1093/aobpla/plac058

**Published:** 2022-12-10

This is a correction to: João Paulo Basso-Alves, Rafael Ferreira da Silva, Gabriel Coimbra, Suzana Guimarães Leitão, Claudia Moraes de Rezende, Humberto Ribeiro Bizzo, Leandro Freitas, Juliana Villela Paulino, Vidal de Freitas Mansano, Heteromorphic stamens are differentially attractive in *Swartzia* (Fabaceae), *AoB PLANTS*, Volume 14, Issue 5, October 2022, plac041, https://doi.org/10.1093/aobpla/plac041

In the originally published version of this manuscript, Table 1 was incorrectly formatted: the data that should be indicated as a white-grey gradient were inadvertently omitted.

This error has now been corrected.

