# Supplementary material for: Heteromorphic stamens are differentially attractive in Swartzia (Fabaceae)
Source: AoB Plants. 2022 Sep 9;14(5):plac041. doi: 10.1093/aobpla/plac041 (PMC9575666; doi:10.1093/aobpla/plac041)

Supplemental material

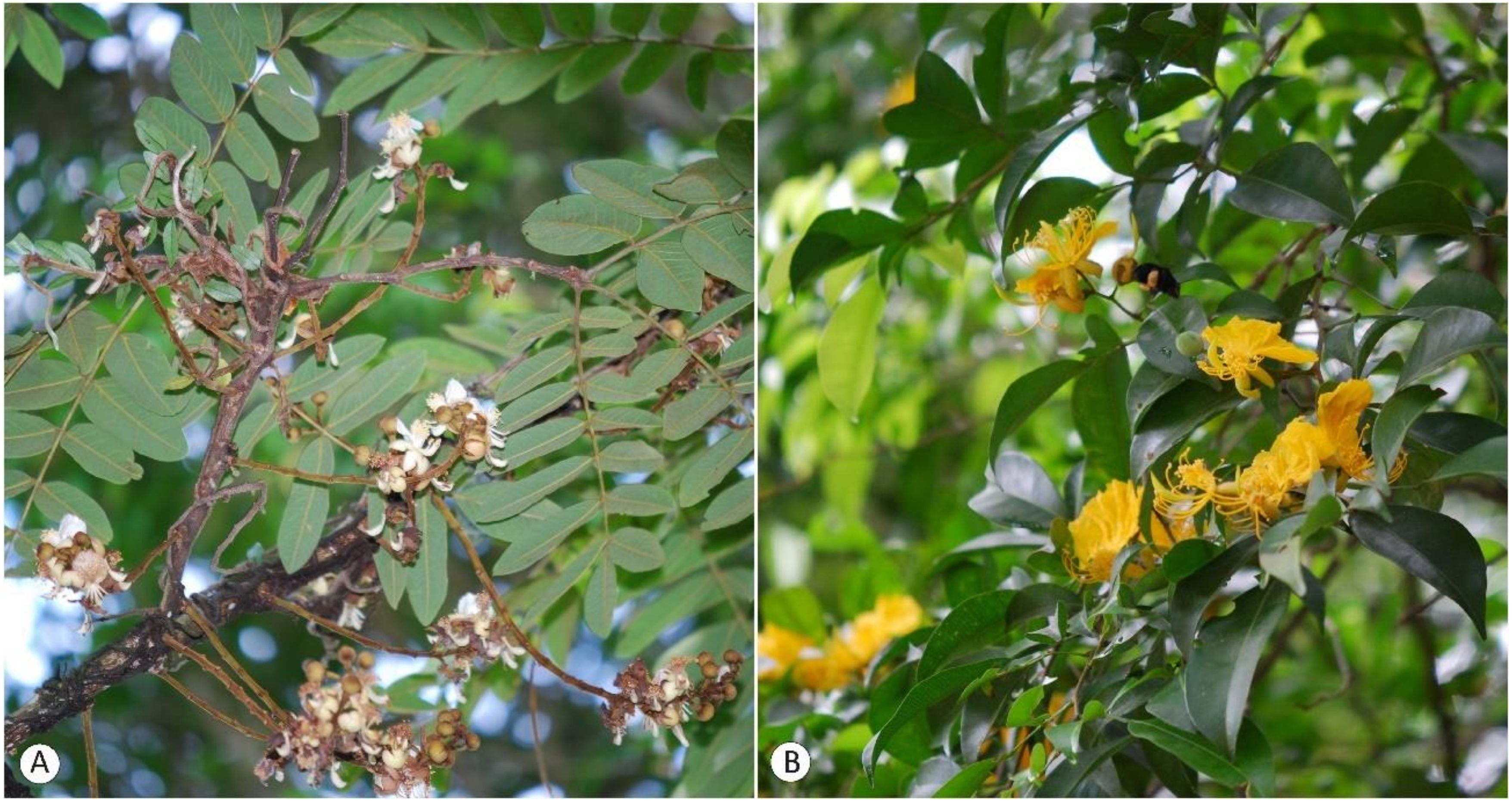

Figure S1. Inflorescences of *Swartzia flaemingii* (A) and *Swartzia simplex* (B).

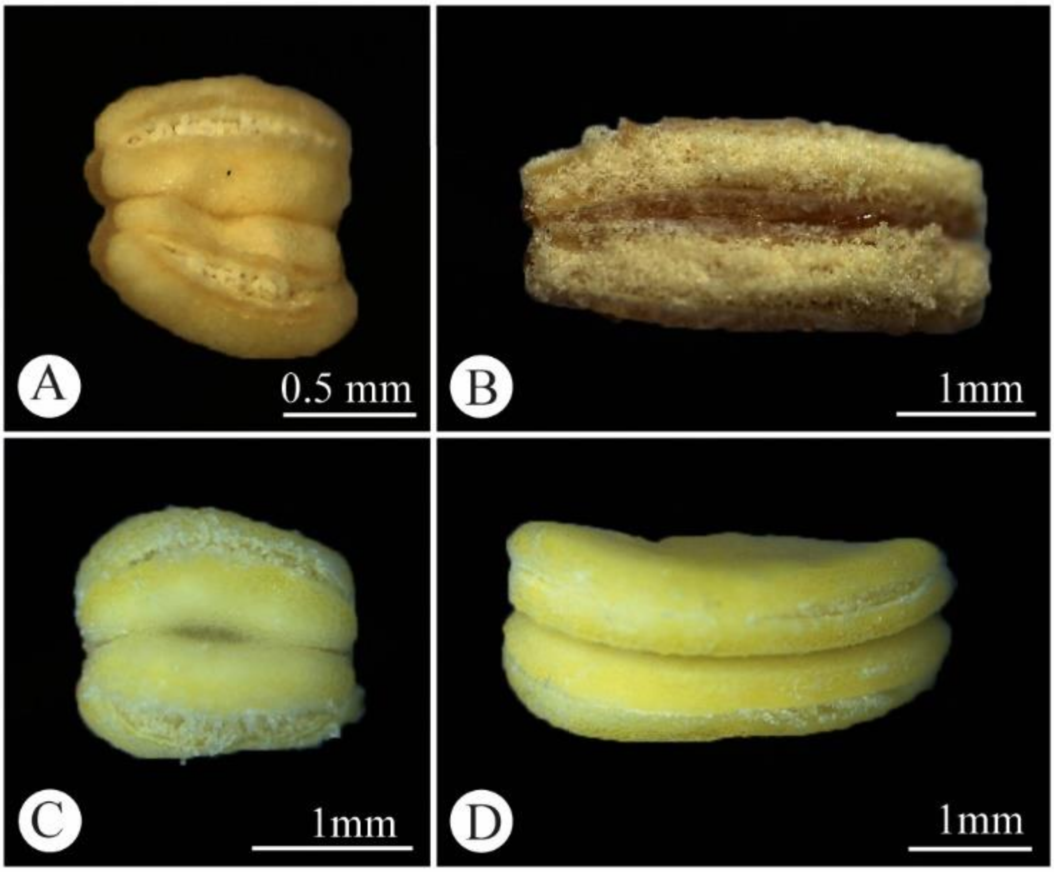

Figure S2. Anthers of *Swartzia*. (A) anther of the smaller stamen of *Swartzia flaemingii*. (B) anther of the larger stamen of *S. flaemingii*. (C) anther of the smaller stamen of *S. simplex*. (D) anther of the larger stamen of *S. simplex*.

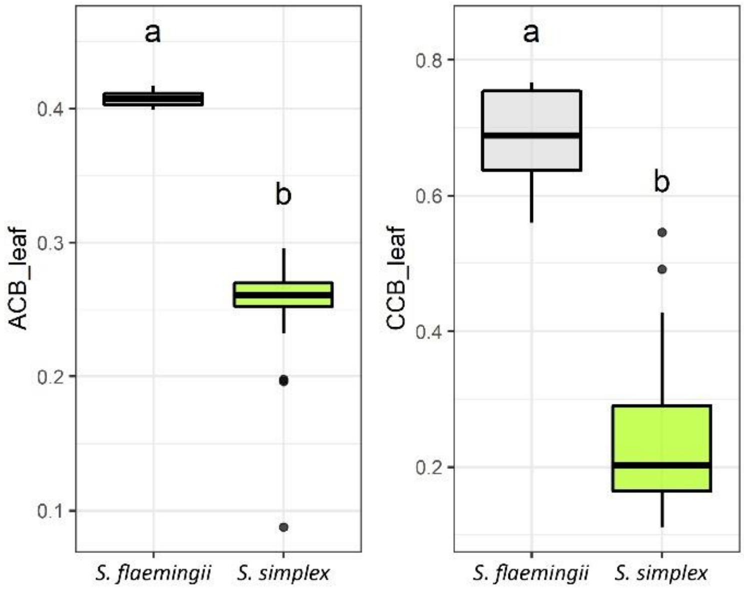

Figure S3. Mean chromatic (CCB) and achromatic (ACB) contrast differences for the petals of *Swartzia flaemingii* and *Swartzia simplex* against a standard green leaf background. Contrasts are given in hexagon units according to the visual system of *Bombus terrestris* L.

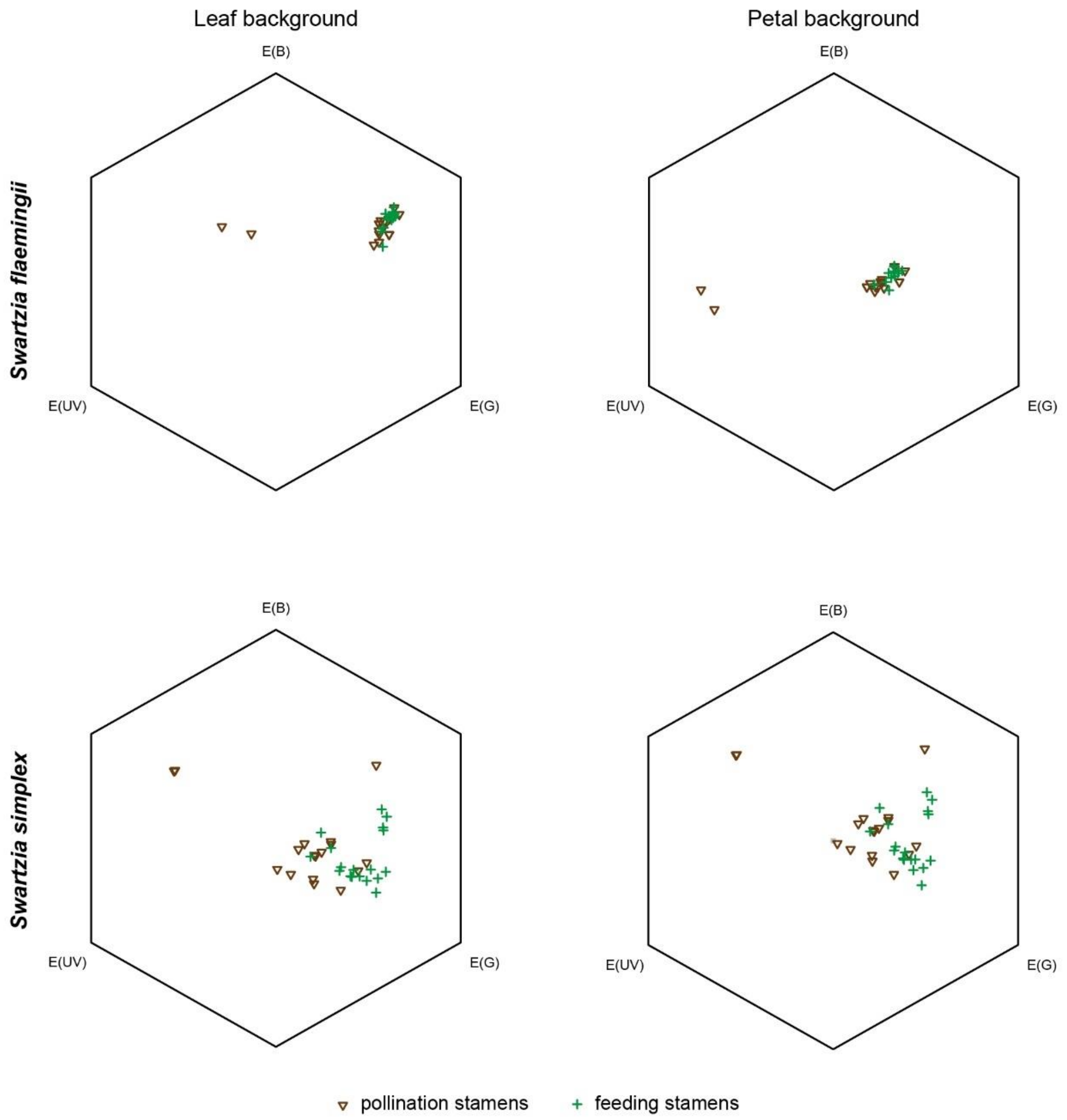

Figure S4. Visual modelling of stamen color using the color hexagon model for *Bombus terrestris* L. The hexagon diagrams represent the relative excitation of each of the three bee photoreceptors (E): ultraviolet (UV), green (G) and blue (B). The points indicate how conspicuous each stamen type is according to the hexagon color model against different backgrounds. Thus, the furthest a point is located from the center of the hexagon, the more visible it is to bee vision, as it stands out from the leaf/petal background (represented by the center of the hexagon). Likewise, the closer two given points are located from each other, the less distinguishable they are in bee vision.

Appendix S5. Chromatograms.

TABLE S1. Constituents and relative percentage of volatile identified in whole flowers (WF) and isolated flower organs (Pet: petals; Ssta: Smaller stamens; Lsta: Larger stamens; Car: carpel) from *Swartzia flaemingii* by HS-SPME-GC-MS.

			Relative percentage (%)				
LRI_{cal}[a]	LRI_{lit}[b]	Compounds	WF	Pet	Ssta	Lsta	Car
792	792	2-propylfuran	0.1		0.1	0.1	
797	792	3-(*E*)-hexenal + hexanal	0.5			2.5	
797	801	hexanal		0.3	9.8		27.7
844	846	2-(*E*)-hexenal	0.3	0.5	25.4	17.3	5.3
853	850	3-(*Z*)-hexen-1-ol	22.6	17.1	6.2	15.1	3.1
860	859	2-(*Z*)-hexen-1-ol		4.6	1.3	5.5	
866	863	*n*-hexanol	9.0	2.9	3.6	5.3	2.5
884	889	2-heptanone	0.2		0.4	0.2	0.2
897	894	2-heptanol	0.2				0.6
903	907	(*E*,*E*)-2,4-hexadienal			1.7	0.4	
925	921	tricyclene		0.1			0.2
928	930	tetrahydro citronellene				0.1	0.2
938	945	α-fenchene					0.3
996	1000	decane		0.3			
1001	1003	ethyl-3-(*E*)-hexenoate + 3-(*Z*)-hexenyl acetate			1.5		
1003	1004	3-(*Z*)-hexenyl acetate	5.5	10.4		2.6	
1005	1007	hexyl acetate	3.0	2.9			
1019	1024	limonene	0.2				0.4
1028	1032	(*Z*)-β-ocimene	0.1	0.3			0.1
1038	1032	(*E*)-β-ocimene	0.7	3.1			
1074	1067	*cis*-linalool oxide	0.5	0.5			1.7
1093	1095	linalool	5.7		0.5	1.3	16.7
1107	1104	α-fenchocamphorone	0.5				1.3
1136	1142	3-(*Z*)-hexenyl isobutanoate	1.6			0.3	
1140	1147	hexyl isobutanoate	0.3			0.1	
1180	1184	3-(*Z*)-hexenyl butanoate	8.9	6.6	0.6	1.2	0.1
1184	1191	hexyl butanoate	2.5	0.7		0.1	
1184	1190	methyl salicylate					0.4
1192	2000	dodecane	0.3	0.7	0.4	0.5	0.6
1222	1229	3-(*Z*)-hexenyl-2-methyl-butanoate	5.6	8.4	0.1	0.9	1.1
1227	1233	*n*-hexyl-2-methyl-butanoate	2.3	4.1	0.2	0.7	0.6
1294	1294	1-tridecene	0.6	0.8	0.5	0.9	0.9
1371	1378	3-(*Z*)-hexenyl hexanoate	2.1	0.2	0.2	0.3	
1474	1469	*n*-dodecanol	4.5	1.7	7.2	5.6	4.5
1488	1493	α-zingiberene			0.3	0.2	
1494	1500	*n*-pentadecane	3.4	1.2	6.0	4.4	3.7
1502	1505	(*E*,*E*)-α-farnesene	1.0		2.9	2.0	0.1
1520	1528	(*E*)-*iso*-γ-bisabolene			0.1	0.1	
1558	1561	(*E*)-nerolidol			0.1		
1666	1667	6-(*Z*)-pentadecen-2-one	0.7	0.6	2.5	1.6	1.0
1677	1671	*n*-tetradecanol	7.2	13.7	14.1	11.7	10.9

[a]Linear Retention Indices relative to C_8-C_{26} n-alkanes on the HP-5MS capillary column.

[b]Linear Retention Indices from Adams (2007).

Chromatograms – *Swartzia flaemingii*

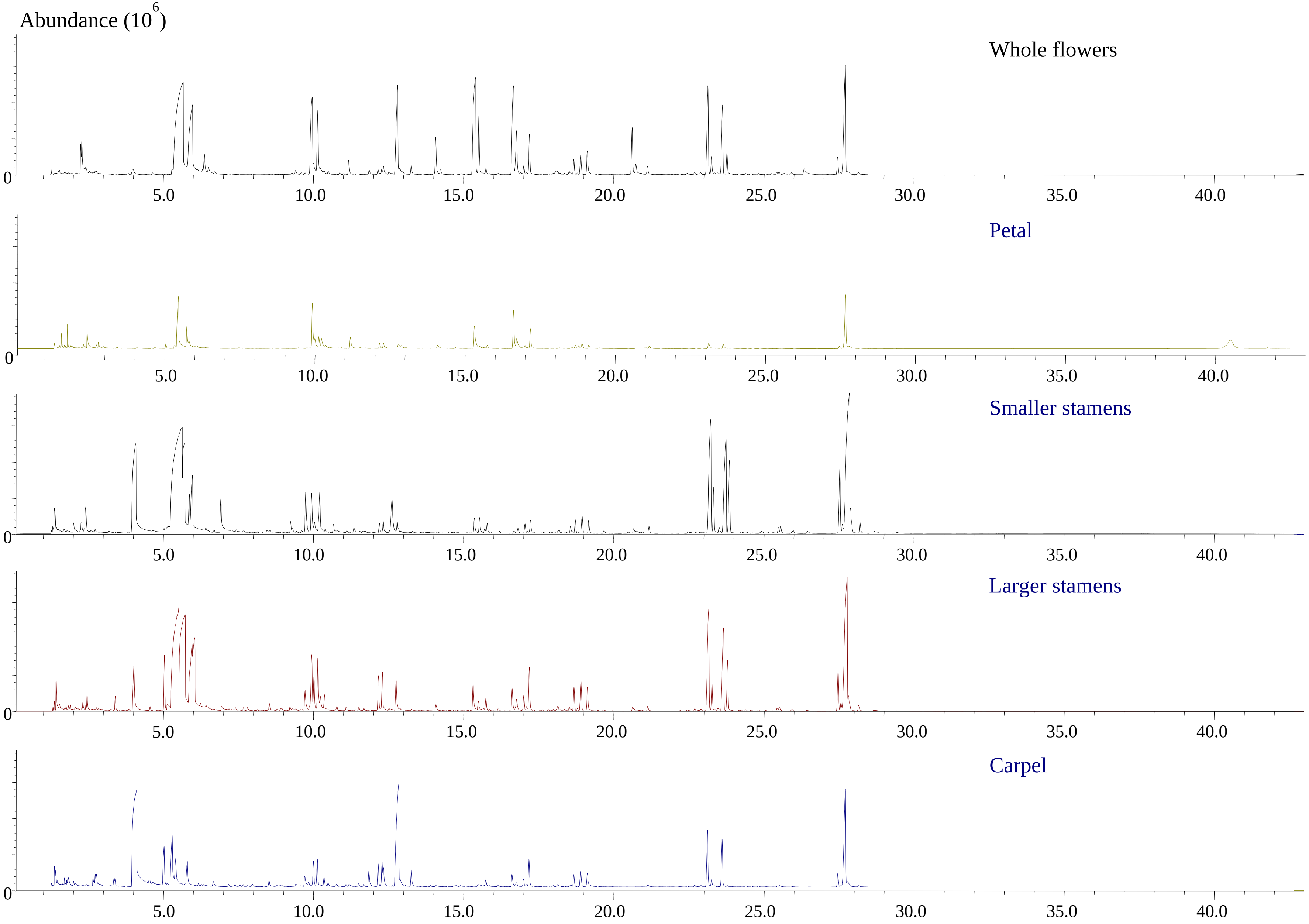

TABLE S2. Constituents and relative percentage of volatile identified in whole flowers (WF) and isolated flower organs (Pet: petal; Ssta: Smaller stamen; Bsta: Larger stamen; Car: carpel) from *Swartzia simplex* by HR-SPME-GC/MS.

			Relative percentage (%)				
LRI_{cal}[a]	LRI_{lit}[b]	Compounds	WF	Pet	Ssta	Lsta	Car
708	711	2-ethoxy ethanol	5.5	4.5			25.3
798	801	hexanal	15.7	15.4	17.5	19.9	15.1
847	846	2-(*E*)-hexenal	8.5	12	8.9	14.9	13.2
866	863	*n*-hexanol	1.8	0.5	2.4	1.0	1.2
953	952	benzaldehyde	3.3	4.6	1.6	1.5	0.8
988	984	2-pentylfuran	1.2	1.0	1.6	0.8	0.5
1018	1020	*p*-cymene	0.1	0.1			
1023	1024	limonene	1.0	0.9	0.8	0.5	0.6
1036	1036	benzene acetaldehyde	1.1	0.4	0.7	0.4	1.4
1046	1044	(*E*)-β-ocimene	1.2	0.1	0.3	0.2	0.9
1054	1049	2-(*E*)-octenal	1.2	1.0	0.5	0.9	0.6
1068	1067	*cis*-linalool oxide	0.4	0.4	0.4	0.5	2.4
1078	1084	*trans*-linalool oxide	0.3	0.3	1.2	1.0	6.2
1093	1095	linalool	7.4	9.3	16.5	17.6	1.4
1129	1134	benzeneacetonitrile	4.0	6.4	0.3	3.8	
1147	1150	(*E*,*Z*)-2,6-nonadienal	10.5	9.3	1.4	3.7	4.8
1153	1157	(2*E*)-nonen-1-al	10.5	7.5	0.2	3.3	2.7
1205	1217	β-cyclocitral	0.4	0.4	0.2	0.4	
1220	1227	nerol	0.4	0.9	7.8	0.2	
1293	1289	thymol	0.4	0.2	2.3	1.1	
1421	1428	(*E*)-α-ionone	0.8	0.9	1.0	1.0	
1431	1434	dihydro-β-ionone	0.4	0.6	0.4	0.7	
1453	1460	sesquisabinene	0.2	0.3	0.4	0.1	
1482	1489	(*E*)-β-ionone	2.9	4.5	6.1	6.9	
1491	1490	α-zingiberene	0.1		0.1	0.1	0.1
1505	1505	(*E*,*E*)-α-farnesene	0.2	0.2	0.7	0.2	
1562	1563	(*E*)-nerolidol	2.2	2.7	3.5	0.6	

[a]Linear Retention Indices relative to C_8-C_{26} n-alkanes on the HP-5MS capillary column.

[b]Linear Retention Indices from Adams (2007).

Chromatograms – *Swartzia simplex*

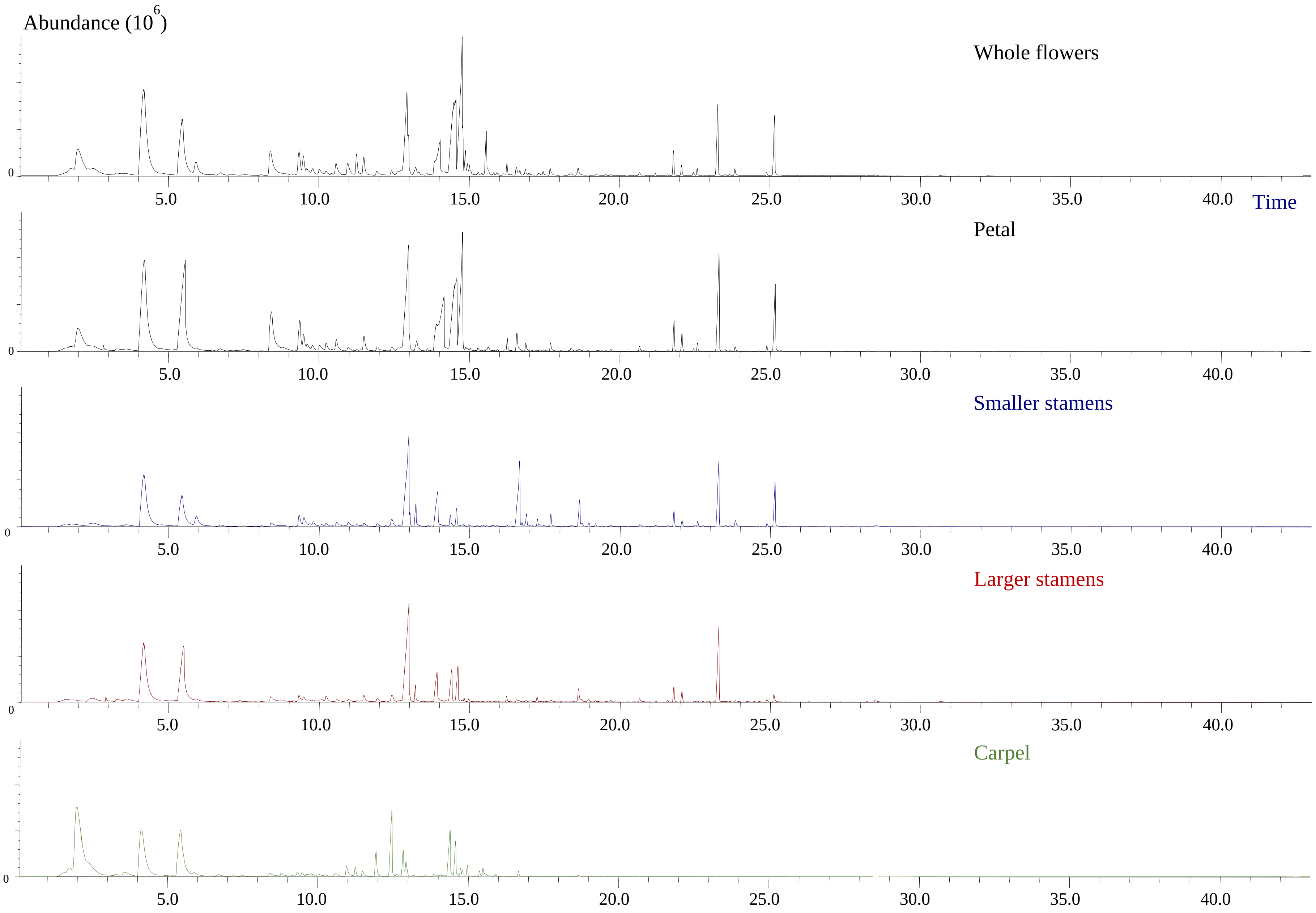

Supplement: plac041_suppl_Supplementary_Material [file plac041_suppl_supplementary_material.pdf]
